# The Influence of Plantar Short Foot Muscle Exercises on the Lower Extremity Muscle Strength and Power in Proximal Segments of the Kinematic Chain in Long-Distance Runners

**DOI:** 10.1155/2019/6947273

**Published:** 2019-01-02

**Authors:** Iwona Sulowska, Anna Mika, Łukasz Oleksy, Artur Stolarczyk

**Affiliations:** ^1^Department of Clinical Rehabilitation, University of Physical Education in Krakow, Poland; ^2^Oleksy Physiotherapy Clinic, Lancut, Poland; ^3^Orthopaedic and Rehabilitation Department, Medical University of Warsaw, Poland

## Abstract

The aim of this study was to evaluate the influence of plantar short foot muscles exercises on the performance of lower extremities in long-distance runners. 47 long-distance runners aged 21-45 years took part in this study. The participants were divided into two groups based on baseline measurement of Foot Posture Index: Group 1 (n=27) with neutral foot and Group 2 (n=20) with slight and increased pronation. The participants performed the exercises daily for 6 weeks. The knee flexors and extensors torque, work, and power on Isokinetic Dynamometer and Running-Based Anaerobic Sprint Test (RAST) were checked at baseline and after 6 weeks of exercises. Higher values of peak torque of knee flexors were observed. This change was statistically significant at high load with angular velocity 90°/s (73.55 Nm at baseline and 89.05 Nm after 6 weeks) and 160°/s (69.40 Nm at baseline and 79.00 Nm after 6 weeks) in Group 2. In both groups higher values of maximum power were noted. Participants in Group 2 achieved lower values in each 35-metre run time and higher values of power. In Group 2 there was significant improvement of total time (35.26 s at baseline and 34.79 s after 6 weeks) compared to Group 1 (37.33 s at baseline and 37.56 s after 6 weeks). Exercises strengthening short foot muscles may improve energy transfer through body segments and increase strength and values of generated power. They should be included as a part of daily training programme of runners. This study was registered in the Australian New Zealand Clinical Trials Registry (ANZCTR). Registration number: ACTRN12615001200572.

## 1. Introduction

The term “core stability” is commonly known in training and rehabilitation. It is a crucial element of motor control for efficient biomechanical function of the musculoskeletal system, energy transfer to the distal parts of the body and minimising joint load. It also allows maintaining correct movement patterns. The activity of core muscles is understood as preprogrammed integration of local single-joint and multijoint muscles to provide stability and initiate motion [1]. The relationship between core muscles and distal segments of the body is widely known. Stability of the core influences the mobility of distal body parts and the pattern of energy generation and transfer from proximal to distal segments. Motor control training for integrated stability and mobility is recommended for rehabilitation and athletic training. Activation of muscles in task-dependent specific patterns based on feed-forward patterns of activation is crucial for sport performance [2].

Anatomy trains, defined as direct fascial connections between adjacent muscular structures within the fascial system, take part in the transfer of energy. Both tension and overloading forces are transferred along these anatomy trains. The foot is a significant element of the four main anatomy trains: the superficial back line, the superficial front line, the lateral line and the spiral line [3]. The plantar intrinsic foot muscles are a part of the superficial back line, and, together with plantar fascia, they control the maintenance of the longitudinal foot arch. They allow bringing both ends of the foot closer to each other, maintaining proper relations between the heads of the first and the fifth metatarsals and the heel bone. In addition, the longitudinal arch of the foot is enhanced by the plantar ligament and the plantar calcaneonavicular ligament, which are located in deeper layers. The dysfunctions within the plantar surface of foot may cause problems, which are transmitted to the upper parts of the anatomy trains. A dysfunction of this part is often associated with hyperextension of the knee, shortening and reduction of flexibility of the hamstring muscles, increased cervical lordosis, and decreased lumbar lordosis [4].

The studies available in literature indicate a relationship between the plantar short foot muscles and the proximal segments with the functional status of the body. It was reported that the strength of these muscles significantly correlated with gait speed [5, 6] and with average step count [7]. While standing and moving, the feet are the points of direct contact between the body and the ground. Therefore, they play an important role in maintaining balance. The results of some studies suggest that the strength of short flexors of toes has an impact on postural control [8]. When the postural demand is increased, for example, during single leg stance, the plantar short foot muscles are significantly more activated than in double leg stance [9].

Core stability training is practiced in many sport disciplines. A stable core is essential for recreational and competitive long-distance runners. Weakness in core stability can lead to decreased efficiency of movement patterns, compensatory movement patterns and overloading tissue as a result [10]. The lack of core stability control has been defined as a risk factor in overuse injuries [10–13]. Similarly, dysfunctions present in distal elements of the kinematic chain may influence proximal segments of the body, e.g., foot pronation may affect the biomechanics of upper joints [14, 15]. Excessive pronation, transferred to internal rotation of the tibia, may cause overloading of the knee joint or other changes in proximal part of the lower extremity [16]. In subjects with increased medial longitudinal arch and tendency to supinated foot posture, lower mobility of the foot and proximal parts is observed [17, 18]. It was reported that the change of foot alignment by an orthosis may influence the bioelectrical activity (sEMG) of muscles in the lower extremities and in the trunk [19].

There are some reports indicating a decrease in biceps femoris sEMG activity after using balanced foot orthotics in runners. The proper posture of the foot and reduced excessive pronation with orthotics may limit internal rotation of the tibia and decrease requirements of the lateral hamstring for controlling this movement [20]. However, other studies have suggested the influence of foot position on the activation of mediolateral hamstrings. Active foot supination selectively activates biceps femoris, and active foot pronation selectively activates the semitendinosus and semimembranosus muscles [21]. What is important, both the plantar short foot muscles and hamstring muscles are the components of the superficial back line. According to the Anatomy Trains paradigm, which assumes myofascial continuity, these structures should be considered globally. A confirmation of this theory may be the immediate improvement of hamstring flexibility observed after a single application of self-myofascial-release technique via a tennis ball on the plantar aspect of the foot [22].

Some authors also observed the effects of foot posture on the other muscle groups. There was a reported relationship between excessive pronation and decreased strength of the long plantar flexor muscles [23] or between foot posture and bioelectrical activity of the vastus lateralis, vastus medialis [24], tibialis posterior [25], tibialis anterior, peroneus longus and brevis, and extensor digitorum brevis [26]. Moreover, the foot posture determines mobility of the foot and influences on the kinematics of lower-extremity joints. The decreased medial longitudinal arch of foot is related to its higher mobility, compared to pes cavus [27–29]. These differences are observed in the movement of the calcaneus during propulsion. In frontal plane, a bigger range of eversion and the total range of motion of the rear foot are observed. Moreover, the mobility of the metatarsus and forefoot is increased and prolonged [26, 30–32].

There are also some reports describing the impact of plantar short foot muscles exercises on its strength [6, 33–36]. However, there are no studies considering the influence of these exercises on lower extremity muscle strength in proximal segments of the kinematic chain. It should be noted that so far the researchers have focused mainly on the influence of core stability training on functioning of the whole kinematic chain. The influence of core stability on distal parts of the body suggests the possibility of reciprocal biomechanical dependence. The aim of this study was to evaluate the influence of exercises of plantar short foot muscles on the performance of lower extremities in long-distance runners, including different types of foot according to the differences between their mobility and the influence on the kinematics of lower extremity joints.

## 2. Materials and Methods

### 2.1. Participants

**Figure 1:**
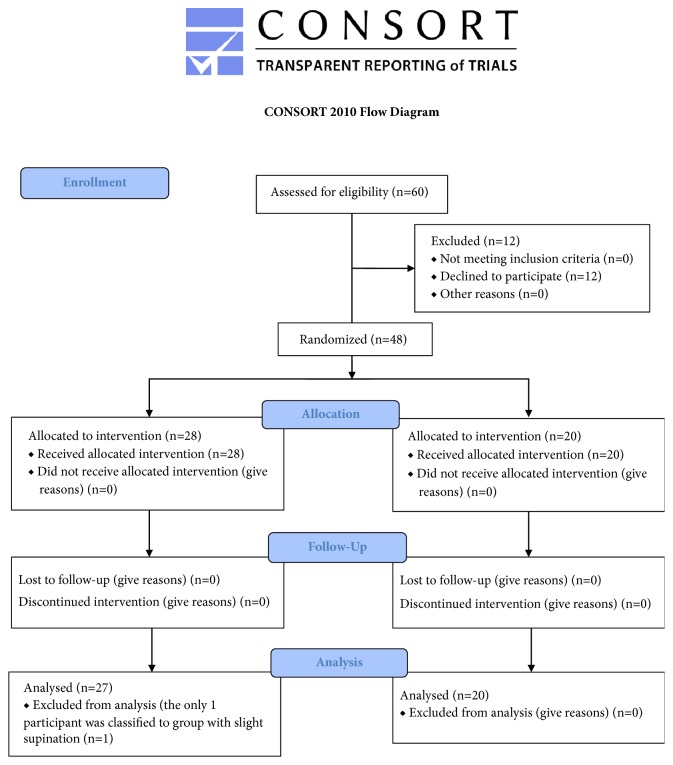
Consort diagram.

47 participants (16 females and 31 males) aged 21-45 years (mean ± SD 32.5 ± 6.81), who run regularly on recreational level with the total distance of 20-100 km per week (mean ± SD 42.19 km ± 18.54 km), participated in this study (Figure 1). Participants were excluded if they had a previous history of acute injury lasting up to six months prior to the enrolment in the study. The other exclusion criteria were as follows: the age less than 20 years old or above 45 years old, the lack of consent to participate in the study, irregular running training, weekly mileage of less than 20 kilometres per week, and visible deformation of the feet.

### 2.2. Procedures

The participants were divided into two groups based on baseline measurement of Foot Posture Index [37]: Group 1 (n=27) rated from 0 to +5 with neutral foot, and Group 2 (n=20) rated from +6 to +10 with slight foot pronation (n=16) and increased foot pronation (n=4). All of the study participants performed exercises of plantar short foot muscles every day for 6 weeks. At this time, they performed current running training, which was monitored by the researchers. This training routine was constant and unchanged throughout the duration of the experiment. All measurements were performed twice: at baseline and after 6 weeks of exercising. The participants were informed in detail about the research protocol and gave their informed consent to participate in the study. The approval of the Ethical Committee of Regional Medical Chamber in Krakow had been obtained before the study.

### 2.3. Research Tools

#### 2.3.1. The Foot Posture Index (FPI-6)

The Foot Posture Index (FPI-6) [37] was used for comprehensive and multidimensional evaluation of the feet. The FPI-6 includes six parts which evaluate particular components of the forefoot and the rearfoot:

1. Talar head palpation,
2. Supra and infra lateral malleolar curvature,
3. Inversion/eversion of the calcaneus,
4. Prominence in the region of the talonavicular joint,
5. Height and congruence of the medial longitudinal arch,
6. Abduction/adduction of the forefoot on the rearfoot.

Each of these parts was rated on a scale from -2 to +2. The negative values indicated supination, while the positive values indicated pronation. The neutral position of the foot was categorized as 0. The total FPI-6 result classified the foot into the following categories:

- from -12 to -5: increased foot supination,
- from -4 to -1: slight foot supination,
- from 0 to +5: neutral foot,
- from +6 to +9: slight foot pronation,
- from +10 to +12: increased foot pronation.

The Foot Posture Index was used to divide participants into two groups.

#### 2.3.2. Isokinetic Dynamometer Prima Doc

The measurement of the knee joint flexors and extensors was performed using Prima Doc isokinetic dynamometer (Easytech, Italy) in a sitting position with the lower extremity flexed in the hip joint to 90°, with the knee axis of rotation concordant with the anatomical axis of the joint. To prevent trunk movements during measurements, the subjects were fastened with a stabilizing strap [38]. The movable arm of the dynamometer was fixed at 1/3 of the distal end of the tibia. Total range of motion (ROM) was set from full extension to full flexion of knee joint. Gravity correction was performed by measuring the torque exerted on the dynamometer resistance adapter by the relaxed, fully extended knee. The tests consisted of 10 maximum isokinetic concentric flexions and extensions of the knee join at each of the 3 angular velocities: 90°/s, 160°/s, and 240°/s with the 30-second rest between them. The following variables were measured: peak torque, total work, average work per repetition and maximum work per repetition, maximum power, and ext/flex ratio. As reported by Larsson et al. [39, 40], the reliability of peak torque was good and ICC ranged between 0.85 and 0.98 for knee extension and 0.88 and 0.97 for knee flexion.

#### 2.3.3. Running-Based Anaerobic Sprint Test (RAST)

The Running-based Anaerobic Sprint Test [41] was used for anaerobic power assessment. The test-retest reliability of the RAST reported by Zagatto et al. [42] was ICC = 0.70-0.97. The RAST consisted of six maximum running efforts on the distance of 35 metres each with 10-second passive rest between all runs. The aim of the test was to evaluate speed, power, and fatigue index. The power in each effort was calculated according to the algorithm:

  Power = total body mass × distance^2^ / time^3^.

These results were used for the calculation of maximum power, minimum power, and mean power. The fatigue index was calculated with the formula:

  Fatigue index = (maximum power – minimum power) / total time of six runs [43].

The time of each 35 metre run was recorded by a system of SmartSpeed PT photocells (Fusion Sport, USA) located at the beginning and at the end of the 35 metre distance. SmartSpeed PT system measures the running time and the reaction time with the accuracy of 0.001 of a second.

### 2.4. Exercise Protocol

All study participants performed the exercises activating the plantar short foot muscles [44, 45]. At baseline, participants were familiarised with the exercises and given written instructions. The exercises were performed by the subjects on a daily basis for 30 minutes for six weeks. Once a week the training was supervised by a physical therapist. The exercise protocol included exercise progression every two weeks by increasing load and level of difficulty and also by using a tennis ball, a stability disc, and band loops. In each exercise participants paid attention to the proper load on the three support points (the heads of the first and the fifth metatarsals and the heel bone). The exercises were performed barefoot.

Vele's Forward Lean was based on the maximum forward lean from standing position. The exercise was performed with arms alongside the body, with feet shoulder width apart, keeping heels on the ground and body in line.

The Reverse Tandem Gait exercise constituted of walking backwards in a straight line with one foot directly behind the other, with arms alongside the body. Initially, the load was put on the metatarsus and then the heel.

In the Short Foot Exercise, participants were instructed to shorten the foot in the anteroposterior direction by attempting to bring the heads of metatarsal bones towards the heel without toe flexion, and then – in shortened position, balance loading of the three support points of the foot. During the exercises, feet were kept on the ground and the toes were relaxed. Increasing the level of difficulty comprised of three variations: sitting position, standing position, and half-squat.

Exercises on the stability disc included several exercises in double-leg standing position and in one-leg standing position.

Exercises with band loops strengthened the muscle-ligament structures of the ankle, including flexion, extension, pronation, and supination with resistance. Before each training, participants performed plantar myofascial self-release with a tennis ball.

### 2.5. Statistical Analysis

The statistical analysis was carried out using the STATISTICA 12.0 Pl software. To assess the normality of variables' distribution Shapiro-Wilk test was performed. The two-way ANOVA with one main factor as between subjects (Group 1 and Group 2) and the other main factor as repeated measure (time: baseline and 6 weeks) was used to determine the significance of the differences of the evaluated variables. Then the Tukey post hoc test was performed. Differences were considered to be statistically significant if the level of the test similarities was lower than the assumed level of significance (p<0.05). A paired t-test power analysis of exercise influence determined that at least 37 subjects were required to obtain a power of 0.8 at a two-sided level of 0.05 with effect size d = 0.8. This analysis was based on data derived from previous literature [6, 33, 46–48].

## 3. Results

### 3.1. Isokinetic Dynamometer Prima Doc

After 6 weeks of exercises of plantar short foot muscles, higher values of peak torque of knee flexors were observed. This change was statistically significant at high load (angular velocity 90°/s and 160°/s) in Group 2 (Table 1).

Compared to baseline after 6 weeks of exercise in both groups, higher values of maximum power of knee joint flexors at each angular velocity were noted. This change was significant in Group 2 at the highest load (90°/s) and at the lowest load (240°/s) (Table 1).

After the exercise programme, the trend for increase in maximum work per repetition, average work per repetition and total work of knee flexor muscles was noted at all velocities (Table 2).

Flexors to extensors ratio increased after 6 weeks of exercises of plantar short foot muscles. This change was statistically significant at high load (90°/s angular velocity) in Group 1 (Table 3). Regarding the knee extensor muscles, none of the parameters showed statistically significant changes after 6 weeks of training (p>0.05). There were also no differences between groups (p>0.05) after the exercise.

### 3.2. Running-Based Anaerobic Sprint Test (RAST)

After 6 weeks of training program, in Group 2 lower values of each 35 metre run time and higher values of power at each runs were observed, compared to Group 1 (Table 4).

**Figure 2:**
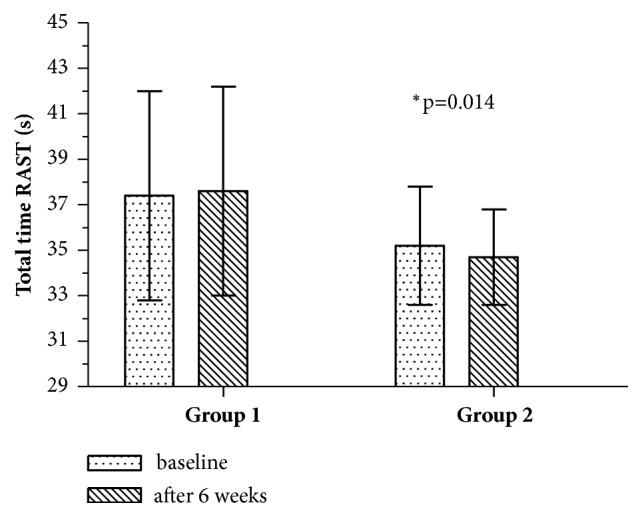
Total time of RAST at baseline and after 6 weeks of exercising. *∗*p: significantly different value.

**Figure 3:**
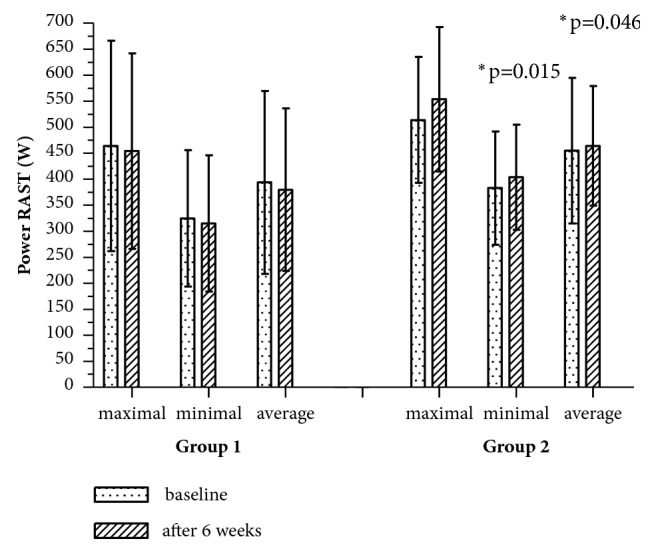
Maximal, minimal, and average power of RAST at baseline and after 6 weeks of exercising. *∗*p: significantly different value.

After 6 weeks of exercise in Group 2, significant improvement of total time of six runs was also observed (Figure 2). Furthermore, runners in this group achieved significantly higher values of minimum and mean power in comparison to Group (Figure 3). There were no significant differences in fatigue index (p>0.05).

## 4. Discussion

The aim of this study was to evaluate the influence of exercises of plantar short foot muscles on the performance of lower extremities in long-distance runners, including different types of foot according to the differences between their mobility and influence on the kinematics of lower-extremity joints. Our study was the first to undertake the subject of impact of exercises of plantar short foot muscles on the lower extremity muscle strength and power in proximal segments of the kinematic chain. The obtained results suggested that this kind of training may improve energy transfer through body segments and increase strength and values of generated power. It is notable that there are no studies dealing with this subject. However, there are some studies indicating the relationship between plantar intrinsic short foot muscles and the upper segments of the body.

Abe et al. [5] evaluated the strength of plantar short foot muscles: flexor digitorum brevis and abductor hallucis, in relation to the anatomical cross-sectional area and physical performance. They observed a significant correlation between the strength of flexor digitorum brevis muscle and its cross-sectional area. In addition, the strength of this muscle had a significant impact on the maximum walking speed of men and women. In our own study, we have evaluated the influence of exercises of plantar short foot muscles on short‐distance running time. After 6 weeks of exercises, runners performed six 35 metre runs at higher speeds.

Goldman et al. [46] reported the effectiveness of a 7-week strength training of toe flexor muscles and athletic performance. The study participants performed isometric plantar flexion contractions with 90% of the maximum voluntary contraction, keeping the metatarsophalangeal joints (MPJ) angle at 25° dorsiflexion. After the training, a significant increase of maximum MPJ and ankle plantar flexion moments, and improvement in horizontal jump distance were observed. The authors suggested that toe flexor muscles may contribute to force generation during lean forward-type movement. The interesting thing is that the improvement was noted only in horizontal jump distance, while no changes were found in vertical jump. Probably, after strength training, toe flexor muscles depressed the toes against the ground more effectively; therefore, the jumpers were able to lean forward more and flatten their take-off angle during horizontal jump. Our research seems to confirm this thesis. The more effective lean forward during sprint start position may be the reason of the improvement observed in RAST test after 6 weeks of training.

In another study, Abe et al. [7] evaluated the influence of daily physical activity on the toe grasping strength and knee extensor strength. They observed that the absolute and relative toe grasping strengths were significantly greater in the group with high intensity of physical activity (with average step count ≥8,000 steps/day) than in the low-intensity group. However, there were no statistically significant differences in absolute and relative knee extension strengths between groups. In our own study, there was also no significant effect of training of plantar short foot muscles on knee extensor muscle strength. After 6 weeks of training programme, a significant improvement was noted only in the performance of knee flexors. It may suggest that hamstring muscles were more susceptible to exercises of plantar short foot muscles, with both of them being parts of the same anatomy train.

Hashimoto and Sakuraba [6] studied the effectiveness of 8-week training on strength of foot flexor muscles. The interphalangeal and metatarsophalangeal joint flexing exercises with resistance were performed with 200 repetitions, once per day, three times per week. After training, they observed significantly higher values of foot flexor muscle strength and improvement in vertical jump, one-leg long jump, and 50 metre run time tests. In our own study, improvement in running test was also observed, although the training was shorter, and the exercises activated only short foot muscles without flexor digitorum longus muscles.

In our previous study [49], the impact of foot exercises on foot posture and fundamental movement patterns in long-distance runners were evaluated. In this study, runners performed two kinds exercises of plantar short foot muscles. After 6 weeks of training, statistically significant improvements in Foot Posture Index (FPI-6) and in Functional Movement Screen (FMS) test, which estimates the quality of the fundamental movement patterns, were observed. It was demonstrated that exercises of plantar short foot muscles significantly modified the foot posture and reduced tendency to pronation. Moreover, beneficial effects on the quality of functional movement patterns in deep squat and active straight leg raise (ASLR) tests were noted. It was suggested that this kind of exercise in addition to strengthening of short foot muscles also positively affected proximal body parts, improving hip mobility, neuromuscular control of hip movement in relation to pelvis, and control of load transfer from toes to heel. The relationship between short foot muscles and proximal parts of the body was also confirmed by the results of our current study. We have hypothesised that the applied exercise, through interaction within myofascial chains, may enhance strength, work, and power in knee flexors and performance in running tests.

Plantar intrinsic foot muscles are a component of the superficial back line. According to the paradigm of Anatomy Trains, the myofascial structures participate in energy transfer through body parts. Tension and overloads are transmitted along them [3]. However, in the literature there are no studies reporting the influence of exercises of plantar short foot muscles on lower extremity muscle strength in proximal segments of the kinematic chain. There are only some studies which partially describe this issue with the relationship between the foot posture and the activity of lower extremity muscles.

Snook et al. [23] conducted a study with isokinetic ankle joint assessment in subjects with neutral foot and with pronation. In the group with pronation, they observed decreased concentric ankle plantar flexion peak torque values in comparison to the group with neutral foot. They explained these results biomechanically, as pronation makes foot less rigid and, therefore, generates less torque [50, 51]. On the other hand, Hintermann and Nigg [16] indicated that excessive foot pronation influences internal rotation of the tibia and may cause overloading of the knee or other changes in proximal parts of the kinematic chain. The authors explained that while walking or running the movement associated with the calcaneus eversion pronation induces internal rotation of the tibia through the talus. At the same time, pelvis rotates externally in relation to the supporting leg, initiating external rotation of the femur. In this way, two forces acting in opposite directions affect the knee joint. Excessive transfer of the eversion movement of the calcaneus to the tibia may be associated with potential knee overload. This situation occurs when foot pronation exceeds physiological range of motion.

However, optimum range of foot pronation is a beneficial mechanism, which provides shock absorption and fine ground contact [16, 52]. In our study, just among runners with foot pronation, an improvement of the evaluated parameters was observed after 6 weeks of foot exercises. Runners with a slight tendency towards pronation have better results in knee flexor strength and in the RAST test, in comparison to the runners with neutral or slightly supinated foot posture. Williams et al. [24] compared leg stiffness between two groups of runners: with high arches and with low arches, with the use of kinetic and kinematic parameters. Also, the bioelectric activity of the following muscles was evaluated: biceps femoris, vastus lateralis, lateral head of the gastrocnemius, and tibialis anterior. They have observed significantly higher leg stiffness and significantly higher knee stiffness in runners with high arches and tendency to supination than in subjects with low arches. Moreover, the high-arched subjects demonstrated decreased knee flexion excursion during stance phase while running, and a significantly earlier onset of vastus lateralis than the low-arched runners. Based on these results, they suggested that excessive supination and high medial longitudinal arch of foot may reduce shock absorption capability and thereby increase the risk of injury.

Gray and Basmaijan [25] evaluated muscles of the lower leg and foot: tibialis anterior and posterior, peroneus longus, flexor hallucis longus, abductor halluces, and flexor digitorum brevis. They reported that subjects with flat-arched foot posture presented increased muscle bioelectrical activity during walking, especially in the stance phase. They suggested that the increased activity of these muscles may be a response to the reduced stability and weakened ligament system in the flat-arched foot posture.

The results from their study were supported also by other authors. Hunt and Smith [26] reported an increased muscle activity during walking in pes planus group. These muscles (tibialis anterior, soleus, gastrocnemius, peroneus longus and brevis, and extensor digitorum longus) were activated with delay, compared to those with neutral foot. Probably in pes planus foot those muscles are actively engaged in longitudinal arch support when body weight is transferred to the forefoot.

In all of the above studies, the authors evaluated only the muscles of the lower leg and foot. In current literature, there are only few reports describing the relationship between foot and thigh muscles. Nawoczenski and Ludewig [20] reported a significant decrease in biceps femoris electromyographic activity in runners, after applying foot orthotics, which changed foot alignment towards neutral position. The proper foot posture and the reduced excessive pronation limited internal rotation of tibia and decreased the requirements of the lateral hamstring for controlling this movement.

Another study suggests the impact of foot position on the activation of medial and lateral hamstring muscles. Lynn and Costigan [21] showed that active foot supination increased the activity of biceps femoris during lower limb exercises (knee flexion and extension with resistance), while active foot pronation selectively activated the semitendinosus and semimembranosus muscles. The researchers recommended applying these results in the training and treatment of imbalance between medial and lateral hamstring muscles.

Grieve et al. [22] evaluated the influence of foot plantar self-myofascial release (SMR) technique with a tennis ball on hamstrings and lumbar spine flexibility. The results indicated an immediate effect of 2 minutes of SMR on the relaxation of hamstrings and lumbar spine muscles. The authors, based on the Anatomy Trains paradigm, suggested that working only on the distal part of superficial back line we may affect the flexibility of its upper parts. In our study, runners also used the SMR technique with a tennis ball as a warm-up and preparation for foot exercises. Based on the observations of Grieve et al., we have suggested that the foot muscle relaxation technique applied in our study could contribute to a better preparation to training of the other muscles of the superficial back line.

Based on our own results and on studies by other authors, we concluded that there is an existing strong relationship between the foot and the upper segments of the body. This relationship is multifactorial, including both the skeletal system—the impact of tibia rotation on proximal segments of the kinematic chain and the interconnections within myofascial structures. According to the Anatomy Trains paradigm, the foot is a component of four main anatomy trains: the superficial back line, the superficial front line, the lateral line, and the spiral line, which participate in the transfer of energy [3].

The relationship between core stability and performance of distal body segments described in the literature [2, 11] enables us to suggest that there is a biomechanical possibility of force transmission in the opposite direction. Our results supported that thesis. In our study, after 6 weeks of exercises of plantar short foot muscles, strength, work and power of the knee joint flexors were improved. In the Running-based Anaerobic Sprint Test, a tendency to lower values of each run time and higher values of power was noted. These changes were observed in runners with tendency to foot pronation, which suggests their greater susceptibility to this kind of training.

As a limitation of this study it should be noted that the total running distance of the subjects ranged between 20 and 100 km per week, which resulted in some heterogeneity of the group. There were also no participants with excessive foot supination in the study population. Moreover, there was no control group. The results obtained in our study allow us to suggest that the exercises of plantar short foot muscles may have beneficial effects on the performance of lower extremity muscles in long-distance runners. It was observed that this possibility resulted from the influence of strengthening of foot short muscles on proximal segments of the kinematic chain. However, these specific exercises are usually ignored in training routine—not only by runners, but also in other sport disciplines. We recommend that these exercises should be included in training by long-distance runners and their coaches should pay attention to their regularity.

## 5. Conclusions

(1) Higher values of peak torque, power, and work suggest that the applied exercises may have beneficial effect on strength of knee joint flexor muscles.

(2) Exercises strengthening short foot muscles may improve energy transfer through body segments and increase values of generated power.

(3) Based on our results, we can suggest that the applied training programme is effective and should be included as a part of daily training programme of runners.

*Practical Implications*

(1) Exercises of plantar short foot muscles may improve energy transfer through body segments.

(2) Exercises of plantar short foot muscles increase strength and values of generated power of lower extremity muscles.

(3) An optimum training of the plantar short foot muscles in long-distance runners should be incorporated in daily training routine.

## Figures and Tables

**Table 1:** Results of peak torque and maximum power of knee joint flexors in the isokinetic dynamometer measurement at baseline and after 6 weeks of exercising.

Outcome Measure		Group 1	P*∗*	Group 2	P*∗*	P*∗∗*
		$\overset{-}{x}+/-SD$		$\overset{-}{x}+/-SD$		
Peak Torque 90 (Nm)	Baseline	76.00 +/- 30.61		73.55 +/- 21.23		0.990
	6 Week	81.11 +/- 28.67	0.732	89.05 +/- 25.03	**0.047**	0.754

Peak Torque 160 (Nm)	Baseline	62.64 +/- 23.13		69.40 +/- 14.75		0.713
	6 Week	68.56 +/- 20.64	0.533	79.00 +/- 25.82	**0.019**	0.361

Peak Torque 240 (Nm)	Baseline	55.57 +/- 17.00		56.95 +/- 16.72		0.991
	6 Week	59.85 +/- 16.08	0.414	59.63 +/- 13.58	0.836	0.999

Max. Power 90 (W)	Baseline	95.85 +/- 42.09		98.70 +/- 29.82		0.994
	6 Week	107.81 +/- 41.73	0.347	118.05 +/- 35.90	**0.005**	0.803

Max. Power 160 (W)	Baseline	123.82 +/- 51.14		124.75 +/- 31.91		0.999
	6 Week	143.22 +/- 50.93	0.188	145.66 +/- 51.05	0.245	0.998

Max. Power 240 (W)	Baseline	146.59 +/- 53.50		142.05 +/- 45.38		0.993
	6 Week	157.00 +/- 58.27	0.672	170.35 +/- 70.91	**0.025**	0.860

*p: p-value.*

*∗ between baseline and 6 week.*

*∗∗ between study groups*.

**Table 2:** Results of maximum work per repetition, average work per repetition and total work of knee flexor muscles in the isokinetic dynamometer measurement at baseline and after 6 weeks of exercising.

Outcome Measure		Group 1	P*∗*	Group 2	P*∗*	P*∗∗*
		$\overset{-}{x}+/-SD$		$\overset{-}{x}+/-SD$		
Max Work x Rep 90 (J)	Baseline	81.96 +/- 38.54		86.60 +/- 28.56		0.965
	6 Week	87.48 +/- 33.91	0.786	91.65 +/- 28.01	0.882	0.974

Max Work x Rep 160 (J)	Baseline	70.74 +/- 31.26		76.65 +/- 24.95		0.874
	6 Week	73.30 +/- 22.13	0.969	80.32 +/- 26.26	0.943	0.805

Max Work x Rep 240 (J)	Baseline	58.15 +/- 26.63		61.40 +/- 23.71		0.966
	6 Week	62.11 +/- 21.01	0.846	65.35 +/- 21.79	0.896	0.966

Avg Work x Rep 90 (J)	Baseline	57.04 +/- 28.87		63.30 +/- 27.39		0.875
	6 Week	64.22 +/- 29.10	0.561	71.20 +/- 26.63	0.605	0.835

Avg Work x Rep 160 (J)	Baseline	40.90 +/- 20.51		44.15 +/- 14.75		0.942
	6 Week	48.22 +/- 20.08	0.297	50.19 +/- 21.09	0.591	0.987

Avg Work x Rep 240 (J)	Baseline	39.31 +/- 18.68		39.60 +/- 15.47		0.999
	6 Week	42.48 +/- 17.90	0.845	43.14 +/- 19.67	0.860	0.999

Total Work 90 (J)	Baseline	572.19 +/- 290.36		632.30 +/- 273.42		0.888
	6 Week	644.70 +/- 291.84	0.553	712.45 +/- 266.92	0.593	0.848

Total Work 160 (J)	Baseline	409.12 +/- 205.30		441.45 +/- 146.82		0.952
	6 Week	482.15 +/- 200.00	0.294	502.07 +/- 210.51	0.584	0.988

Total Work 240 (J)	Baseline	393.34 +/- 184.87		396.25 +/- 155.10		0.999
	6 Week	425.44 +/- 179.27	0.839	431.63 +/- 197.29	0.859	0.999

*p: p-value.*

*∗ between baseline and 6 week.*

*∗∗ between study groups*.

**Table 3:** Results of flexors to extensors ratio in the isokinetic dynamometer measurement at baseline and after 6 weeks of exercising.

Outcome Measure		Group 1	P*∗*	Group 2	P*∗*	P*∗∗*
		$\overset{-}{x}+/-SD$		$\overset{-}{x}+/-SD$		
Flx/Ext Ratio 90 (%)	Baseline	42.78 +/- 8.42		47.15 +/- 8.36		0.475
	6 Week	52.15 +/- 12.90	**0.002**	52.11 +/- 10.10	0.315	0.999

Flx/Ext Ratio 160 (%)	Baseline	44.56 +/- 10.45		52.07 +/- 7.54		0.164
	6 Week	51.78 +/- 14.72	0.117	55.45 +/- 14.16	0.794	0.737

Flx/Ext Ratio 240 (%)	Baseline	46.67 +/- 10.71		49.17 +/- 6.26		0.906
	6 Week	53.07 +/- 16.46	0.151	52.80 +/- 13.46	0.720	0.999

*P: p-value.*

*∗ between baseline and 6 week.*

*∗∗ between study groups*.

**Table 4:** Results of RAST at baseline and after 6 weeks of exercising.

Outcome Measure		Group 1	P*∗*	Group 1	P*∗*	P*∗∗*
		$\overset{-}{x}+/-SD$		$\overset{-}{x}+/-SD$		
Run 1–time (s)	Baseline	5.92 +/- 0.70		5.56 +/- 0.41		0.191
	6 Week	5.94 +/- 0.75	0.997	5.48 +/- 0.33	0.373	0.064

Run 2–time (s)	Baseline	5.98 +/- 0.78		5.62 +/- 0.43		0.233
	6 Week	5.97 +/- 0.77	0.999	5.58 +/- 0.37	0.630	0.174

Run 3–time (s)	Baseline	6.23 +/- 0.80		5.87 +/- 0.53		0.281
	6 Week	6.28 +/- 0.80	0.914	5.80 +/- 0.37	0.545	0.084

Run 4–time (s)	Baseline	6.32 +/- 0.81		5.99 +/- 0.48		0.351
	6 Week	6.37 +/- 0.83	0.912	5.93 +/- 0.40	0.589	0.145

Run 5–time (s)	Baseline	6.46 +/- 0.85		6.12 +/- 0.49		0.357
	6 Week	6.57 +/- 0.86	0.573	6.01 +/- 0.38	0.256	**0.049**

Run 6–time (s)	Baseline	6.41 +/- 0.86		6.11 +/- 0.47		0.471
	6 Week	6.42 +/- 0.87	0.999	5.98 +/- 0.39	0.154	0.162

Power of run 1 (W)	Baseline	455.23 +/- 192.00		502.81 +/- 122.01		0.777
	6 Week	447.49 +/- 190.08	0.975	545.01 +/- 145.30	0.112	0.220

Power of run 2 (W)	Baseline	429.08 +/- 173.61		512.29 +/- 151.40		0.335
	6 Week	440.45 +/- 185.94	0.948	518.49 +/- 139.23	0.822	0.392

Power of run 3 (W)	Baseline	398.33 +/- 183.78		430.72 +/- 110.41		0.882
	6 Week	376.21 +/- 155.15	0.649	460.58 +/- 117.14	0.237	0.231

Power of run 4 (W)	Baseline	360.59 +/- 139.31		428.01 +/- 140.55		0.356
	6 Week	362.98 +/- 155.09	0.999	430.00 +/- 103.83	0.935	0.361

Power of run 5 (W)	Baseline	355.72 +/- 158.66		399.11 +/- 117.10		0.688
	6 Week	329.40 +/- 134.66	0.464	413.80 +/- 104.93	0.471	0.150

Power of run 6 (W)	Baseline	348.74 +/- 137.38		400.43 +/- 117.68		0.546
	6 Week	355.75 +/- 151.10	0.963	421.15 +/- 104.22	0.314	0.341

*p: p-value.*

*∗between baseline and 6 week*.

*∗∗between study groups*.

## Data Availability

The datasets used and analysed during the current study available from the corresponding author on reasonable request.
